# Chinese gut microbiota and its associations with staple food type, ethnicity, and urbanization

**DOI:** 10.1038/s41522-021-00245-0

**Published:** 2021-09-06

**Authors:** Jing Lu, Li Zhang, Qixiao Zhai, Jianxin Zhao, Hao Zhang, Yuan-Kun Lee, Wenwei Lu, Mingkun Li, Wei Chen

**Affiliations:** 1 State Key Laboratory of Food Science and Technology, Jiangnan University, Wuxi, China; 2 School of Food Science and Technology, Jiangnan University, Wuxi, China; 3 Key Laboratory of Genomic and Precision Medicine, Beijing Institute of Genomics, Chinese Academy of Sciences, and China National Center for Bioinformation, Beijing, China; 4 National Engineering Research Center for Functional Food, Jiangnan University, Wuxi, China; 5 International Joint Research Laboratory for Pharmabiotics & Antibiotic Resistance, Jiangnan University, Wuxi, China; 6 Department of Microbiology & Immunology, Yong Loo Lin School of Medicine, National University of Singapore, Singapore, Singapore; 7 University of Chinese Academy of Sciences, Beijing, China; 8 Center for Excellence in Animal Evolution and Genetics, Chinese Academy of Sciences, Kunming, China

**Keywords:** Microbiome, Clinical microbiology

## Abstract

The gut microbiota could affect human health and disease. Although disease-associated microbiota alteration has been extensively investigated in the Chinese population, a nationwide Chinese gut microbiota baseline is still lacking. Here we performed 16 S rRNA gene sequencing on fecal samples from 2678 healthy Chinese individuals, who belonged to eight ethnic groups and resided in 63 counties/cities of 28 provinces. We identified four enterotypes, three of which were enriched for *Prevotella*, *Bacteroides,* and *Escherichia*, respectively, whereas the fourth one had no dominant genus. By assessing the association between the gut microbiota and 20 variables belonging to six categories, geography, demography, diet, urbanization, lifestyle, and sampling month, we revealed that geography explained the largest microbiota variation, and clarified the distinct patterns in the associations with staple food type, ethnicity, and urban/rural residence. Specifically, the gut microbiota of Han Chinese and ethnic minority groups from the same sites was more alike than that of the same ethnic minority groups from different sites. Individuals consuming wheat as staple food were predicted to have more microbial genes involving in glucan 1,3-beta-glucosidase and *S*-adenosyl-l-methionine biosynthesis than those who consumed rice, based on functional prediction. Besides, an appreciable effect of urbanization on decreased intra-individual diversity, increased inter-individual diversity, and increased proportion of the *Bacteroides* enterotype was observed. Collectively, our study provided a nationwide gut microbiota baseline of the Chinese population and knowledge on important covariates, which are fundamental to translational microbiota research.

## Introduction

The human intestine harbors a special diverse microbial ecosystem, with an estimated 150–400 bacterial species reside in our gut^1^. The gut microbiota provides substantial benefits to our health by forming a barrier against pathogens, producing bioactive metabolites, and regulating immunological functions. The homeostasis of the gut ecosystem is maintained by some core species that are generally shared among different individuals, and the gut microbiota in healthy adults is relatively stable in the absence of strong influencing factors (e.g., dietary changes or antibiotic treatment)^2,3^.

The imbalance of the gut microbiota (i.e., dysbiosis) is associated with many diseases, e.g., inflammatory bowel disease, obesity, allergies, and autoimmune diseases^4^. Plenty of microbial components have been revealed to involve in a series of pathologies by extensive disease-targeted microbiota researches, and can thus, in theory, serve as biomarkers. For example, fecal microbial markers for screening colorectal cancer have been widely studied^5^. However, the translation of microbiota research into clinical practice is still limited by multiple challenges, especially the difficulty in the precise classification of “healthy” microbiota, which requires comprehensive knowledge of the microbiota variation and covariates of an average, healthy population. A study based on 7009 individuals from 14 geographic districts in one province of China demonstrated that microbiota-based metabolic disease models developed in one location could not be extrapolated to other locations, and the efficiency of interpolated models decreased as geographic scale increased^6^. This emphasized the influence of geography on gut microbiota composition and disease model application, whereas the microbiota variation on a larger geographic scale across China is yet to be explored.

Besides geography, many gut microbiota covariates have been uncovered, including diet, lifestyle, ethnicity, socioeconomic status, medication, and genetics^7,8^. For example, stratification of the gut microbiota (termed enterotypes) has been associated with diet, especially the intake of fibers and carbohydrates^9^; urbanization has been associated with increased inter-individual variation and loss of species with high potential for fiber degradation^10^; taxa that were differentially abundant across ethnicities have been proposed to be associated with chronic diseases^11,12^. To date, most population-level studies investigating gut microbiota covariates have focused on the western population (Europe and the USA) and a few in Israel, Japan, and China^13^, but rarely in Africa, South America, and other regions of Asia.

There are various food styles in China, and people living in different regions show great varieties in their diet. Moreover, there are 56 ethnic groups in China, which have distinct characteristics in diet, lifestyle, custom, and culture. Therefore, the diversified gut microbiota is expected in the Chinese population. Meanwhile, as a result of an unprecedented speed and scale of urbanization, Chinese is undergoing rapid change in lifestyle, and the dietary habit is shifting towards a western-style diet. Specifically, more high-fat and high-protein foods are consumed while fewer grains are taken, which might significantly change the gut microbiota as observed in other developing countries. Although a couple of studies have been conducted to investigate the gut microbiota characteristics in China, these studies either focused on limited regions or recruited a small number of participants, and a nationwide gut microbiota survey is still missing^6,14,15^.

To characterize the gut microbiota diversity in the Chinese population and investigate microbiota-associated variables, we collected feces of 2678 individuals without apparent diseases (referred to as “healthy”), which underwent 16 S ribosomal RNA (rRNA) gene sequencing (V3–4 region). A questionnaire including information on demography, diet, and lifestyle was carried out, which enables an in-depth analysis of factors associated with the Chinese gut microbiota.

## Results

### Overview of cohort and data

**Fig. 1:**
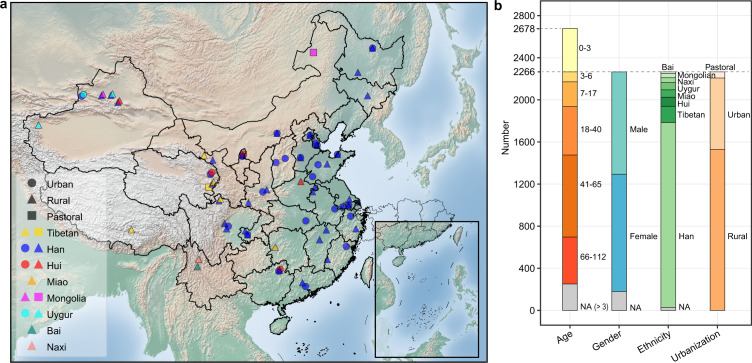
Overview of cohort. **a** Geographic distribution of sampling sites, ethnic groups, and urbanization status. Sampling sites were mapped using the Matplotlib Basemap Toolkit. **b** Cohort composition with respect to age, gender, ethnicity, and urbanization status. See also Figure S1.

We recruited 2678 healthy volunteers (male 1144, female 973) from 63 counties/cities of 28 provinces, including 2167 Han Chinese (1755 with age over 3 and 412 with age under 3), 487 individuals from seven ethnic minority groups (Tibetan, 156; Hui, 107; Miao, 73; Uygur, 70; Naxi, 46; Mongolian, 41; Bai, 18), and 24 individuals without ethnicity information (Fig. 1a, b). Fecal samples were collected following a standardized procedure (see Methods for details). Meanwhile, 20 phenotypical and environmental variables were collected via questionnaires or national annals, and classified into six categories: geography, demography, diet, urbanization, lifestyle, and sampling month (Supplementary Data 1). The gut microbiota was profiled by sequencing the variable region 3–4 (V3–4) of 16 S rRNA gene, with a median read number of 27,638 per sample (range 10,000–236,350). The reads were clustered into 14,364 zero-radius operational taxonomic units (ZOTUs), and 56.64% of these ZOTUs (accounting for 86.48% of the total reads) were assigned to 444 genera belonging to 24 phyla.

It is known that a stable gut microbiota resembling that of adults is established at age 3^16^. In our data, we observed a strong positive correlation between the alpha diversity and age in children under age 3 (Shannon index, *R*^2^ = 0.37, observed ZOTUs, *R*^2^ = 0.31, Faith’s phylogenetic diversity (Faith’s PD), *R*^2^ = 0.35, *p* < 2.2e-16), but not in other age groups (age 3–17, 18–65 or 66–112, *p* > 0.05, Supplementary Fig. 1). Thus, only 2266 individuals with ages 3–112 (median 46) were included in the following analyses.

### The gut microbiota composition of the Chinese population and associated covariates

**Fig. 2:**
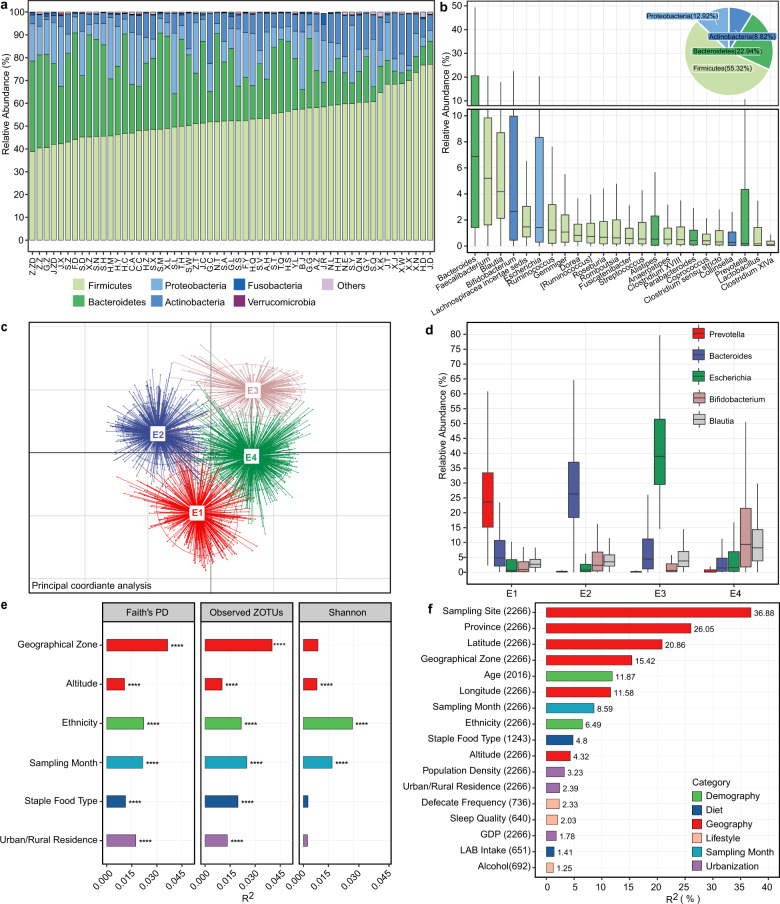
The gut microbiota composition of the Chinese population and associated covariates. **a** Relative abundances of the top six phyla at each sampling site. **b** Relative abundances of the 24 core genera, which presented in >90% of individuals with an average relative abundance >0.1%. The pie chart shows the phylum-level microbial composition of the cohort. LAB: lactic acid bacteria. **c** PCoA plot showing four enterotypes. **d** Relative abundances of representative genera of enterotypes. **e** Covariates associated with microbiota alpha diversity. Covariates having adjusted *R*^2^ > 0.01 and ****p < 0.0001 (simple linear regression) with at least one alpha index were shown. **f** Covariates associated with microbiota beta diversity estimated by JSD distance. The effect size was calculated with envfit (vegan), and covariates with *p*.adj < 0.05 were shown. The number of samples was indicated in brackets following each covariate. In boxplots, the center line represents the median, box limits represent upper and lower quartiles and whiskers represent 1.5× interquartile range. See also Figure S2.

Firmicutes, Bacteroidetes, Proteobatcteria, and Actinobacteria were the four most abundant bacterial phyla in all samples (Fig. 2a, b). A total of 24 genera were observed in >90% of samples with average relative abundances >0.1% (the core microbiota, Fig. 2b). Eighteen of these genera overlapped with the core gut microbiota of 2008 healthy Chinese individuals who resided in Guangdong province^6^ (Supplementary Data 2); seven of them overlapped with the top nine most abundant fecal genera in another Chinese cohort, which included 314 healthy individuals from nine provinces^14^; ten of them overlapped with the top 20 fecal genera discovered by the Human Microbiome Project^17^. We further stratified the microbiota into four enterotypes using the clustering method described by Arumugam et al. ^18^. We identified driving genera by random forest algorithm (area under the curve (AUC) for receiver operating characteristic (ROC) curve: 0.99, Supplementary Fig. 2a), obtaining *Prevotella* enterotype (E1, *n* = 443), *Bacteroides* enterotype (E2, *n* = 732), *Escherichia* enterotype (E3, *n* = 251), and mixture enterotype (E4, *n* = 840) (Fig. 2c, d). E1 and E2 are two well-recognized enterotypes, whereas unlike Firmicutes (most prominently *Ruminococcus*) being the third enterotype in most previous studies^19^, E3 was distinguished by an overrepresentation of *Escherichia* (a genus belonging to family Enterobacteriacease, phylum Proteobacteria), which has rarely been reported^20^. Besides, E4 showed no dominant genus but a mixture of a few relatively abundant genera, including *Bifidobacterium* and *Blautia* etc.

Covariates associated with the alpha diversity of gut microbiota were first investigated with simple linear regressions. Faith’s PD and Observed ZOTUs were significantly correlated with geographical zone (including ten zones differing in climate, topography, etc., Supplementary Fig. 2b), altitude, staple food type, urban/rural residence, ethnicity, and sampling month (adjusted *R*^2^ > 0.01, *p* < 0.0001, Fig. 2e). The correlations were validated in multiple linear models incorporating all six covariates, except that with altitude (Supplementary Data 3). This reflected the dependence of altitude, which was different among ethnic groups (Supplementary Data 1). In addition, a simple linear model using only Han individuals did not support the correlation between alpha diversity and altitude either.

Meanwhile, the gut microbial community structure (beta diversity, estimated by Jensen-Shannon divergence (JSD)) was significantly correlated with 17 covariates as evaluated by envfit^21^ (*p*.adj < 0.05, Fig. 2f). Geographic factors (sampling site, province, latitude, and geographical zone) explained the largest variance, followed by age, sampling month, ethnicity, staple food type, urbanization, and other geographic factors (Supplementary Fig. 2c, d for Bray–Curtis and unweighted UniFrac distances). To further explore the correlation between geographic location and the gut microbiota, we applied the Mantel test on the microbial JSD matrix and geographic distance matrix, and found a significant correlation between them (*p* = 0.03, Supplementary Fig. 2e), suggesting that the gut microbiota change gradually in proximal locations. Detailed analysis of these associations was conducted in the following sections.

### Association between staple food type and the gut microbiota

**Fig. 3:**
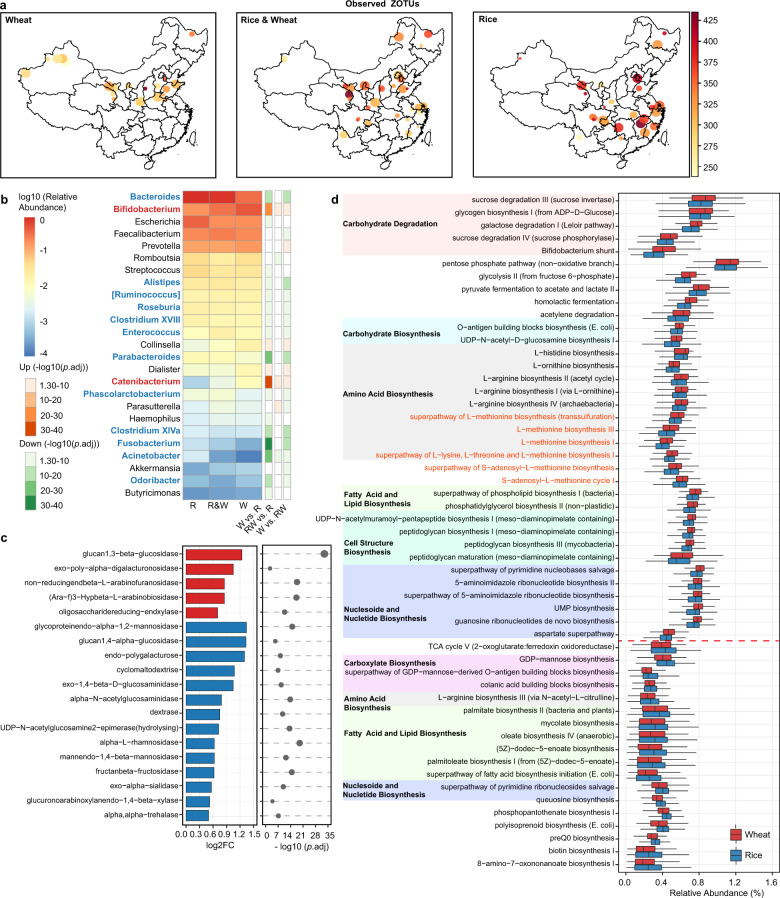
Differences in the microbiota alpha diversity, composition, and functional potential between populations consuming different staple food. **a** Observed ZOTUs. Each dot represents a sampling site; the color of the dot indicates the median value in each site; the diameter of the dot is proportional to the number of samples in each site and is fixed for sites with >15 samples. **b** Differential genera between the three groups. Genera with *p*.adj <0.05 from DESeq2 analyses were shown. Genera highlighted in red or blue indicate higher abundances associated with wheat or rice intake, which were detected with two DESeq2 models. *R* rice, *W* wheat. **c** Differential glycosidase between the Rice and Wheat group. Glycosidase with log2FC > 0.5 and *p*.adj < 0.05 from Mann–Whitney tests were shown. **d** Differential pathways between the Rice and Wheat group. Pathways were grouped by the functional category, and those with higher abundance in the Wheat group were placed on the upper part of the plot. Pathways related to l-methionine and *S*-adenosyl-l-methionine biosynthesis were highlighted in red. Pathways with log2FC > 0.1 and *p*.adj < 1e-10 from Mann–Whitney tests were shown. In boxplots, the center line represents the median, box limits represent upper and lower quartiles and whiskers represent 1.5× interquartile range. Rice: *n* = 417, Wheat: *n* = 549, Rice & Wheat: *n* = 277. See also Figure S3.

Samples were assigned into three groups according to the dominant staple food regularly taken, i.e., rice (white rice), wheat (white flour of common wheat), and rice & wheat. Owing to the requirements in temperature, precipitation, and sunshine duration for different grain crops, wheat was mainly grown in northern China, whereas rice was cultivated more widely^22^. Consumption of the two grains also showed similar geographic distribution (Fig. 3a). Intriguingly, the alpha diversity indices including Faith’s PD, Shannon index and Observed ZOTUs were significantly higher in individuals/regions consuming more rice (*p* < 0.01, Fig. 3a, Supplementary Fig. 3a–c).

Bacterial genera differing between each two groups were identified using DESeq2 analysis with adjustment for age and gender (*p*.adj < 0.05, Fig. 3b). *Bifidobacterium* and *Catenibacterium* were enriched in individuals consuming wheat and wheat & rice when comparing to individuals consuming rice. The abundances of these two genera decreased gradiently in the three groups, indicating that there might be a dose-effect associated with wheat intake. The association between *Bifidobacterium* and wheat intake is consistent with previous observations that the abundance of *Bifidobacterium* was decreased when taking diets with low wheat content, e.g., gluten-free diet, low-gluten diet, and low FODMAP (fermentable oligosaccharides, disaccharides, monosaccharides, and polyols) diet^23–28^. Twelve genera were enriched in individuals consuming rice and rice & wheat when comparing to individuals consuming wheat, of which *Bacteroides*, *Parabacteroides*, a butyrate producer *Clostridium XIVa* and an opportunistic pathogen *Fusobacterium* were the most significant ones (log2FC > 1, *p*.adj < 1e-10). However, no difference was observed in the enterotype composition among the three groups.

To explore the metabolism capacity of gut microbiota affected by distinct staple foods, enzyme commission (EC) numbers and MetaCyc pathways were inferred with PICRUSt2^29^. Considering that common wheat flour contains more dietary fiber than white rice (2–3% vs. 0.7–2% of dry matter) and the fiber components differ significantly between the two grains^30,31^, we specifically focused on 69 ECs belonging to glycosidase (EC 3.2.1). The abundances of 19 glycosidases differed between the Wheat and Rice group (log2FC > 0.5, *p*.adj < 0.05, Fig. 3c). The Wheat group showed dramatically higher glucan 1,3-beta-glucosidase (EC 3.2.1.58), in line with the fact that beta-glucan containing beta-(1->3)-linkages exists in wheat but not rice. A total of 53 pathways differed moderately between the Wheat and Rice group (log2FC > 0.1, *p*.adj < 1e-10, Fig. 3d). First, the Wheat group was distinguished by the higher potential of a few carbohydrate degradation pathways, as well as glycolysis, pentose phosphate pathway, and lactate/acetate fermentation. Second, the Wheat group showed increased capacity for biosynthesis of amino acids including l-methionine, *S*-adenosyl-l-methionine (SAM) and l-arginine etc. Of note, SAM is widely adopted as a therapy for liver disease, depression, and osteoarthritis^32,33^. Besides, the Wheat group was associated with a higher potential of housekeeping functions including cell structure biosynthesis and nucleic acid processing. These findings indicate that the staple food type and possibly related dietary habits may alter the metabolism capacity of gut microbiota.

### Association between ethnicity and the gut microbiota

**Fig. 4:**
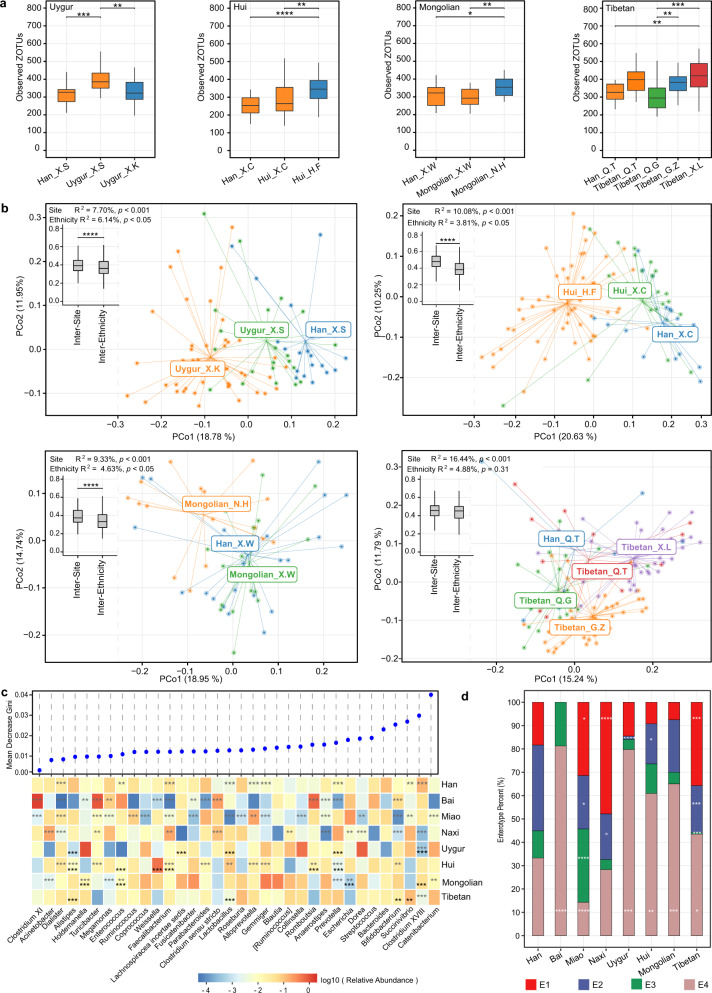
Gut microbiota characteristics of different ethnic groups. **a** Observed ZOTUs of different ethnic groups per sampling site. The labels of *x* axis indicate ethnic groups followed by sampling sites. *p.adj < 0.05, **p.adj < 0.01, ***p.adj < 0.001, Mann–Whitney test. **b** PCoA plot based on JSD. Inserted boxplots show the inter-site distance for samples belonging to the same ethnic groups, and inter-ethnicity distance for samples from the same sampling sites; ****p < 0.0001, Mann–Whitney test. Corresponding *R*^2^ and *p* values from PERMANOVA tests were also shown. **c** Differences in the abundances of predominant genera between ethnicity groups. Genera were ordered by mean decrease in Gini from random forest models classifying ethnic groups. Genera with average relative abundance >1% and presence in >50% of the samples in at least one ethnic group were shown. Asterisks represent *p*.adj values from DEseq2 models; the gray ones indicate comparisons between one ethnic group and the rest groups (and adjusted for sampling sites), while the black ones indicate comparisons between each of the ethnic minority groups (Hui, Mongolian, Tibetan, and Uygur) and their accompanying Han samples from the same sampling sites; **p.adj < 0.01, ***p.adj < 0.001. **d** Percentage of enterotypes in each ethnic group. *p < 0.05, **p < 0.01, ***p < 0.001, Fisher’s exact test comparing ethnicity minority grou*p*s to Han. In boxplots, the center line represents the median, box limits represent upper and lower quartiles and whiskers represent 1.5× interquartile range. **c**–**d** Han: *n* = 1755, Bai: *n* = 16, Miao: *n* = 70, Naxi_ *n* = 46, Uygur: *n* = 69, Hui: *n* = 87, Mongolian: *n* = 40, Tibetan: *n* = 154. See also Figure S4.

Among eight ethnic groups included in this study, Tibetan had the highest alpha diversity, whereas Bai had the lowest alpha diversity (Supplementary Fig. 4a). In addition, the gut microbial community structure differed between ethnic groups (*R*^2^ = 4.00%, *p* < 0.001, permutational multivariate analysis of variance (PERMANOVA) based on JSD, supplementary Fig. 4b). However, as some ethnic groups reside in specific geographic locations, it was hard to partial out the geographic effect. In our study, four ethnic minority groups, namely, Uygur, Hui, Mongolian, and Tibetan, each had not only samples collected from different sites, but also accompanying Han Chinese samples collected from the same sites, which enabled us to distinguish the effect of ethnicity on microbiota from that of geography. The gut microbiota richness (Observed ZOTUs) differed between samples belonging to the same ethnic groups but from different sites (at least 200 km apart) for all four ethnic minority groups. In contrast, the microbiota richness of different ethnic groups from the same sites did not show significant differences except that between Uygur and Han (*p*.adj < 0.05, Fig. 4a, supplementary Fig. 4c, d for Shannon index and Faith’s PD). As to microbiota beta diversity, clustering by sampling site and ethnic group were both distinguishable on the principal coordinate analysis (PCoA) plot (*p* < 0.05 except for Tibetan vs. Han from the same site, PERMANOVA based on JSD, Fig. 4b). The inter-site distance for samples belonging to the same ethnic groups was greater than the inter-ethnicity distance for samples from the same sites (*p* < 0.0001 for Uygur, Hui, and Mongolian), and correspondingly, the sampling site explained larger variance in the gut microbiota than the ethnic group (*R*^2^ of PERMANOVA: Uygur, 7.70% vs 6.14%; Hui, 10.08% vs 3.81%; Mongolian, 9.33% vs 4.63%; Tibetan, 14.44% vs 4.88%) (Fig. 4b). These observations indicated that both geography and ethnicity could affect the gut microbiota, but the former is likely to have a stronger effect.

The genus-level microbiota profile of each ethnic group showed a distinct pattern in relative abundances, and we were able to distinguish different ethnic groups using the random forest model (AUC of the model, 0.88; Miao, 0.94; Uygur, 0.93; Bai, 0.93; Tibetan, 0.92; Naxi, 0.91; Hui, 0.82; Han 0.80; Mongolian, 0.80; Fig. 4c, Supplementary Fig. 4e). *Catenibacterium* contributed the most power to the classification, and the representative genera of enterotypes, Bacteroides (E2), Escherichia (E3), and Prevotella (E1), ranked 5th, 8th, and 9th of the contributing genera, respectively. Correspondingly, the enterotype composition differed among ethnic groups (Fig. 4d). Comparing to Han, Miao, Naxi, and Tibetan had higher proportions of E1; Miao, Naxi, Uygur, Hui, and Tibetan had lower proportions of E2; Miao had higher proportions while Tibetan had lower proportions of E3; all ethnic minority groups except Naxi had higher proportions of E4 (*p* < 0.05, Fisher’s exact test). We further applied DESeq2 models to detect ethnicity-specific genera by comparing one ethnic group to the rest, with adjustment for the confounding factor sampling site; for Uygur, Hui, Mongolian, and Tibetan, we also compared each of them to their accompanying Han samples from the same sampling sites. Differential genera (*p*.adj < 0.05) detected by both models included a lower level of *Clostridium XVIII* in Uygur, lower levels of *Prevotella*, *Fecalibacterium,* and *Alistipes,* whereas a higher level of *Romboutsia* in Hui, and higher levels of *Holdemanella* and *Enterococcus* whereas a lower level of *Escherichia* in Mongolian (Fig. 4c). Of note, the one-versus-rest comparison could be affected by the uneven sample size across ethnic groups.

### Association between urbanization and the gut microbiota

**Fig. 5:**
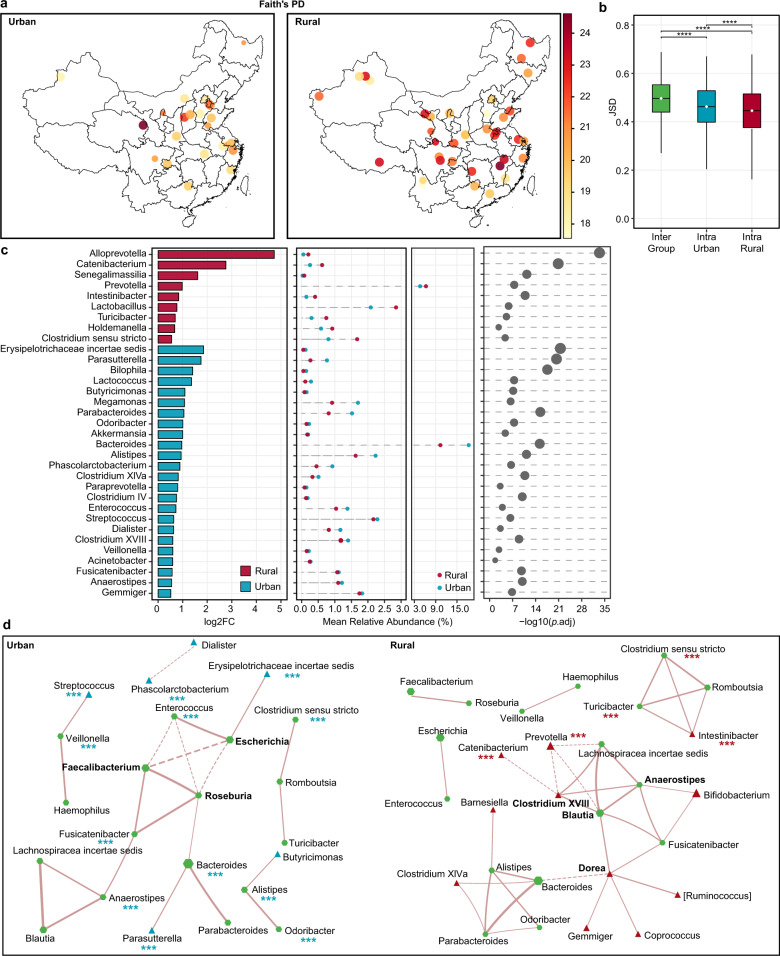
Differences in the microbiota diversity, composition, and network between urban and rural populations. **a** Faith’s PD. Each dot represents a sampling site; the color of the dot indicates the median value in each site; the diameter of the dot is proportional to the number of samples in each site and is fixed for sites with more than 15 samples. **b** Inter- and intra-group JSD. The intra-group distance was calculated per sampling site. ****p < 0.0001, Mann–Whitney test. The center line of the boxplot represents the median, box limits represent upper and lower quartiles and whiskers represent 1.5× interquartile range. **c** Differential genera were detected with the DESeq2 model. Genera with *p*.adj < 0.05 were shown. **d** Co-occurrence network of genera. SparCC correlations with *r* > 0.35 and *p* < 0.01 were shown. Each node re*p*resents a genus; the size of the node is proportional to the median relative abundance of the corresponding genus; the green, red, and blue colors represent the shared genus, rural-specific genus, and urban-specific genus, respectively. The solid and dotted lines represent positive and negative correlations, respectively, and the thickness of the edge is proportional to the *r* value. Red and blue asterisks indicate differential genera shown in **c**, ***p.adj < 0.001. Urban: *n* = 637, rural: *n* = 1530. See also Figure S5.

By comparing the gut microbiota of 1530 residents from 38 rural sites of 24 provinces and 637 residents from 22 urban sites of 18 provinces, we found that Faith’s PD of rural residents was higher than that of urban residents, but Observed ZOTUs and Shannon index did not differ (Fig. 5a, Supplementary Fig. 5a–c). It suggested that urbanization might not affect the non-phylogenetic richness and evenness of the gut microbiota, but instead decrease its phylogenetic richness. Meanwhile, the overall gut microbiota composition differed between rural and urban residents (PERMANOVA based on JSD, R^2^ = 1.63, *p* < 0.01). Of note, the intra-group microbiota dissimilarity evaluated by JSD was higher in the urban residents (*p* < 0.001, Fig. 5b).

The microbiota communities were compared using DESeq2 analysis to determine genera segregating urban and rural populations (*p*.adj < 0.05, Fig. 5c). Of the 33 differential genera, the top two most abundant (mean relative abundance >4.6%) ones were *Bacteroides* that were enriched in the urban population, and *Prevotella* that was enriched in the rural population. Besides, a few low-abundant genera (relative abundance <0.8%) showed more significant differences between the two groups (log2FC > 1.5, *p*.adj < 1e-20), including *Erysipelotrichaceae incertae sedis* and *Parasutterella* that were higher in the urban population, as well as *Alloprevotella* and *Catenibacterium* that were higher in the rural population.

Besides differences in the microbial composition mentioned above, we wonder if microbial interactions were also altered by urbanization, and thus constructed co-occurrence networks by SparCC for urban and rural populations separately (Fig. 5d). The rural population showed a larger network than the urban population (27 nodes with 36 edges vs 23 nodes with 23 edges), and random subsampling of the rural group to the equal number of samples in the urban group confirmed such a difference. Eighteen of the nodes were shared by the two networks, but only ten of the edges were shared, suggesting that correlations between the same microbial pairs were different between the two populations. Hub nodes also differed between the two networks. In the urban population network, two short-chain fatty acids (SCFA) producers, *Roseburia* and *Faecalibacterium*^34^, as well as *Escherichia* connected with more edges, whereas the other three SCFA producers, *Blautia*, *Anaerostipes,* and *Dorea*^34,35^, as well as *Clostridium XVIII* connected with more edges in the rural population network, suggesting different ecological assemblies supporting SCFA production in the gut ecosystem of the two populations.

## Discussion

We have conducted a study on Chinese gut microbiota with a large cohort covering by far the greatest diversity of the healthy population. We found that a series of factors belonging to five categories, i.e., geography, demography, diet, urbanization, and sampling month, explained a substantial proportion of the gut microbiota variation, although the effect sizes of some factors (e.g., ethnicity) were likely under-estimated owing to the uneven number of samples in some subgroups. First, geographic factors showed the strongest signals, including sampling site, geographical zone, altitude etc. Specifically, the gut microbiota of Han Chinese and ethnic minority groups from the same sampling sites was more alike than that of the same ethnic minority groups from different sampling sites, underscoring the importance of considering the geographic location in case-control studies. Although gut microbiota has been widely reported to vary across geography^36^, it is hard to dissect the effect, as geography reflects a mixed effect of lifestyle, long-term diets etc. As to this cohort, we were not able to clarify patterns related to geographical zones, and the effect of altitude was likely linked to minority ethnic groups living in a plateau. Second, we focused on the ethnic group, which represents a highly diverse demographic character of the Chinese population. The Han Chinese and seven ethnic minority groups showed distinct gut microbiota profiles, with some of the variation being attributed to geography and a considerable part remaining significantly explained by the ethnic group. Larger cohorts and targeted design are required to understand the effect of covariates underlying ethnicity (e.g., genetics, custom) on the gut microbiota, and to what extent were these effects homogenized by, for example, co-residing with Han Chinese. Furthermore, our finding highlighted that urbanization was associated with decreased intra-individual diversity and increased inter-individual diversity of the gut microbiota. Previous studies based on one or two provinces of China have shown a similar pattern in the bacterial, fungal, and viral components^15,37,38^, whereas our study based on 28 provinces further validated the vast effect of urbanization on the gut microbiota across China. In addition, although sampling month appeared to be a notable signal, we did not find any specific seasonal pattern in alpha or beta diversity. In contrast, the seasonal rhythm of gut microbiota and its CAZYome diversity has been shown in hunter-gatherers^39^. We speculate that the lack of association between the gut microbiota and season in this cohort is because lifestyle, especially diet, is much less affected by season in the modernized population.

It is well acknowledged that diet alters gut microbial composition and metabolism, but the study on the long-term effect of staple food type on gut microbiome at the population level is still missing^27,40^. The Chinese population mainly consumes two distinct types of staple food, wheat (products made from white flour of common wheat) and rice (boiled white rice). In this study, the wheat-consuming population was predicted to have a remarkably higher level of microbial genes encoding glucan 1,3-beta-glucosidase, the substrate of which only exists in wheat but not rice. This confirmed the validity of not only differences in the amount of wheat intake acquired by questionnaires, but also functional prediction based on 16 S rRNA gene profiles. Further, the predicted increase in the microbial biosynthesis capacity of l-methionine and its major downstream product SAM in the Wheat group is of special interest, owing to the broad involvement of SAM in cognitive and metabolic health^32^. Notably, the alteration of microbial l-methionine biosynthesis as well as the archaeal conversion of SAM has been shown in an intervention study with gliadin (one of the protein fractions of wheat) in mice, which was based on urinary metabolome with ultra performance liquid chromatography-mass spectrometry^41^. Therefore, the possible effect of staple food on SAM biosynthesis may be narrowed down to the effect of the protein component of wheat, gluten, which could trigger celiac disease in 0.06% of the Chinese population^42^. Thus, we speculate that gluten may modulate the health of gluten-tolerant individuals by regulating the gut microbiota, the validity, and mechanism of which warrant further investigation. In addition, since wheat is more popular in north China than in south China, the observed effect of staple food type on gut microbiota could be confounded by geographic locations.

**Fig. 6:**
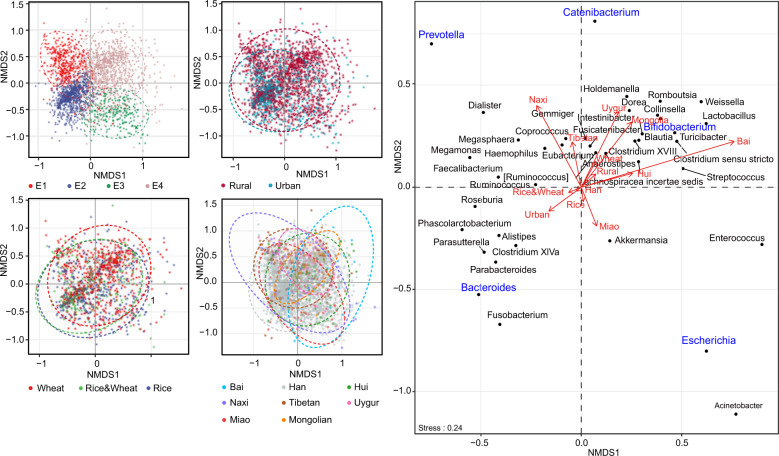
Nonmetric multidimensional scaling plots showing clustering of Chinese gut microbiota by enterotype, urban/rural residence, staple food type, and ethnic group. The ordination was performed with Bray–Curtis distance based on the top 40 genera. Covariates were posteriorly projected onto the plot using envfit (vegan). Ellipses represent 95% confidence.

We identified specific components of the microbiota that were significantly affected by the above factors. The three representative genera of enterotypes, *Bacteroides*, *Prevotella,* and *Escherichia* drove the diversification of the gut microbiota of the Chinese population (Fig. 6). Abundances of the three genera varied in subpopulations consuming different staple food, in different ethnic groups, and in urban vs rural residents. It is likely due to that dietary habits might be the shared covariate underlying these factors, and the close relation between *Bacteroides*/ *Prevotella* and dietary habits especially fiber, protein, and animal fat has been widely shown^19^. Another remarkable genus is *Catenibacterium*, which was found to be more abundant in the population resided in rural areas, and the population consuming wheat as a staple food in this study. It was also the most differential genus among eight ethnic groups in this cohort. *Catenibacterium* was detected in 41% of the population with an average relative abundance of 0.6%. Limited studies on this genus have associated it with diet, but with conflicting results. A few studies showed its association with the Mediterranean diet and low risk of cardiovascular disease^43,44^, whereas the others showed its association with a high-fat, high-sugar diet^45^. These findings address the need for further investigation of *Catenibacterium* in relation to diet and human health. In addition, *Bifidobacterium* was associated with wheat intake, and it also contributed significantly to the differentiation of enterotypes and the eight ethnic groups. Of note, the overall abundance of *Bifidobacterium* in our cohort was considerably higher than that in the AGP cohort and other western cohorts^17,46^, which may further strengthen its effect on health in specific subgroups of the Chinese population.

This study is limited by the absence of comprehensive physiological indices and detailed dietary information. Such information is essential in understanding factors responsible for or affected by the singular gut microbiota characteristics uncovered here, e.g., the enterotype *Escherichia* that was rarely reported in other cohorts, *Catenibacterium* that was enriched in specific subsets of the Chinese population, microbial SAM biosynthesis that possibly exerts an influence on human health. Nevertheless, the obtained profiles have constructed the Chinese gut microbiota baseline, providing information on the microbiota variation, covariates, and the effect size of covariates, which are essential for calculating sample size and statistical power for biomedical studies^47,48^. Moreover, this study has raised attention to considering differences in the microbiota background and confounding factors, including those generally applicable or specific to the Chinese population, in microbiota researches and clinical translations.

## Methods

### The cohort and ethics

The 2678 Chinese participants had no self-reported gastrointestinal tract disorder or any other acute/chronic/recurrent medical conditions (referred to as “healthy”), and they had not taken any antibiotics for at least 3 months prior to participation. All recruited rural or pastoral residents lived a typical farming or pastoral lifestyle away from the metropolitan areas. Fecal samples were collected following a standardized procedure: the participants were informed of detailed instructions, collected samples by themselves, and stored samples in home freezers or iceboxes; samples were transported to the freezer at each sampling site within a day, and further to the research laboratory with cold-chain within 3 days; samples were then well homogenized, aliquoted, and stored at −80 °C until further analyses. The metadata (Supplementary Data 1) was collected via questionnaire, including demographic information (age, gender, ethnicity, BMI, BMI *z* scores), dietary information (staple food type, intake of lactic acid bacteria (LAB)), and lifestyle (defecate frequency, sleep quality, alcohol intake, smoking). Specifically, the ethnic information was confirmed through participants’ Resident Identity cards. BMI *z* scores were calculated for individuals younger than 18 years old using the Zanthro function of STATA package v15.0, based on the World Health Organization Child Growth Standards 2007. Urbanization information (urban/rural/pastoral residence, population density, GDP) was collected from the National Bureau of Statistics of China and China Statistical Yearbook (2017 and 2018). Geographic information (sampling site, province, geographical zone, altitude, latitude, longitude) and sampling month were collected in the meantime. Geographic distances between sampling sites were represented by Vincenty distances, which were computed using the geosphere R package v1.5-10^49^.

The study was approved by the Ethical Committee of Jiangnan University. Written informed consents were obtained from all participants or their legal representatives for minors.

### 16 S rRNA gene sequencing

Microbial DNA was extracted from feces using the MP FastDNA Spin Kit for Feces (MP Biomedicals, Santa Ana, CA, USA) following the manufacturer’s instructions. The V3–4 region of 16 S rRNA gene was amplified by the primers 314 F (CCTAYGGGRBGCASCAG) and 806 R (GGACTACNNGGGTATCTAAT) jointed with a seven-base-pair barcode. The PCR product was purified by the QIAquick Gel Extraction Kit (Qiagen, Hilden, Germany), and sequenced on the Illumina Miseq platform with the Miseq Reagent Kit V3 (Illumina, San Diego, CA, USA, PE300 mode).

### Microbiota data analysis

Paired-end sequencing reads were merged using USEARCH v11.0.667^50^. The reads were de-multiplexed, and barcode and primer sequences were removed with Cutadapt v2.11^51^. All sequences were subjected to quality filtering with a cutoff of maxee 1.0 and de-replicated, and unique sequences with more than seven replicates were clustered to ZOTUs using USEARCH. Taxonomy of ZOTU representative sequences was assigned using the SINTAX algorithm of USEARCH and the adjusted Ribosomal Database Project (RDP) training set v16 (https://github.com/Li-Zhang/rdp_16s_v16_sp_ManualAdjustment) with a cutoff of 0.8. The phylogenetic tree was constructed by inserting ZOTU representative sequences into the 99% Greengenes 13_8 reference tree using the SEPP algorithm^52^ with QIIME2 v2018.10^53^. The functional potential was predicted based on ZOTUs using PICRUSt2^29^, generating EC number and MetaCyc pathway abundances.

For analyzing the microbiota alpha and beta diversity, the ZOTU table was rarefied to 10,000 reads, and observed ZOTUs, Faith’s PD, Shannon index, Bray–Curtis distance, and unweighted UniFrac distance were estimated using QIIME2, whereas JSD was estimated using the phyloseq R package v1.32.0^54^. The microbiota variation explained by environmental variables was evaluated with envfit function of the vegan R package v2.5–6^21^, and the differential clustering of microbial communities was assessed using PERMANOVA with adonis function of vegan. The Correlation between geographic distances and microbial JSD was estimated by Mantel statistic based on Spearman’s rank correlation, using mantel function of vegan.

For the rest of the analyses, total sum scaling was applied to normalize the microbiome data unless otherwise specified. Enterotype analysis was done based on the method described by Arumugam et al.^14^. Samples were clustered with the pam function of the cluster R package v2.1.0^55^. The optimal number of clusters was four according to the Calinski–Harabasz Index, giving an average silhouette coefficient of 0.14. The clustering was visualized on PCoA using the ade4 R package v1.7-15^55^. To identify the driving genera of each enterotype, random forest analysis with ten-time fivefold cross-validation was performed using the randomForest R package v4.6-14^56^. The representative genus *Escherichia*/*Shigella* was referred to as *Escherichia*, considering that all subjects had no symptoms of *Shigella* infection at the time of sampling.

Random forest models with ten times fivefold cross-validation was applied to identify genera distinguishing the eight ethnic groups using the scikit-learn Python package v0.23.1^57^. To even out the number of individuals in each ethnic group, the Han Chinese were randomly downsampled to 6% of the full data set 1000 times. Accordingly, the Mean Decrease in Gini, AUC, sensitivity, specificity, and precision were calculated as the average value from 1000 random forest models.

To examine the microbiota co-occurrence network, correlation analysis was performed on genera (data rarefied to 10,000 reads) using SparCC^58^, and visualized using Cytoscape v3.5.1^59^. Differential genera were detected using the DESeq2 R package v1.29.14^60^, with adjustment for age and gender unless otherwise specified. Genera with mean relative abundance >0.1% and presence in >50% of the samples in at least one group were used for the above analyses unless otherwise specified.

### Statistical analysis

Two-tailed Mann–Whitney test and Fisher’s exact test were used to compare continuous variables and categorical variables, respectively. Multiple comparisons were corrected using the Benjamini–Hochberg false discovery rate algorithm^61^ with a significance level of 0.05 (*p*.adj value).

### Reporting summary

Further information on research design is available in the Nature Research Reporting Summary linked to this article.

## Supplementary information

Supplementary Information

Supplementary Data 1

Supplementary Data 2

Supplementary Data 3

Reporting Summary

## Data Availability

The microbial DNA sequences encoding 16 S rRNA V3–4 region reported in this paper have been deposited in the Genome Sequence Archive in National Genomics Data Center^62^, Beijing Institute of Genomics (China National Center for Bioinformation), Chinese Academy of Sciences, under accession number CRA003616 that are publicly accessible at https://bigd.big.ac.cn/gsa.
