# Supplementary material for: Chinese gut microbiota and its associations with staple food type, ethnicity, and urbanization
Source: NPJ Biofilms Microbiomes. 2021 Sep 6;7:71. doi: 10.1038/s41522-021-00245-0 (PMC8421333; doi:10.1038/s41522-021-00245-0)
Supplement: Supplementary file 1 — Supplementary Information [file 41522_2021_245_MOESM1_ESM.pdf]

The Supplementary materials include:

Supplementary Figures:

Supplementary Figure 1. The association between microbiota alpha diversity and age.

Supplementary Figure 2. The gut microbiota composition of the Chinese population and associated covariates.

Supplementary Figure 3. Microbiota alpha diversity of populations consuming different staple food.

Supplementary Figure 4. Gut microbiota diversity of different ethnic groups.

Supplementary Figure 5. Microbiota alpha diversity of urban and rural populations.

Supplementary Datasets:

Supplementary Data 1. Demographic information and environmental factors of samples in this study.

Supplementary Data 2. Core microbiota of healthy individuals from the Guangdong Gut Microbiome Project.

Supplementary Data 3. Covariates associated with microbiota alpha diversity as estimated by multiple linear regression.

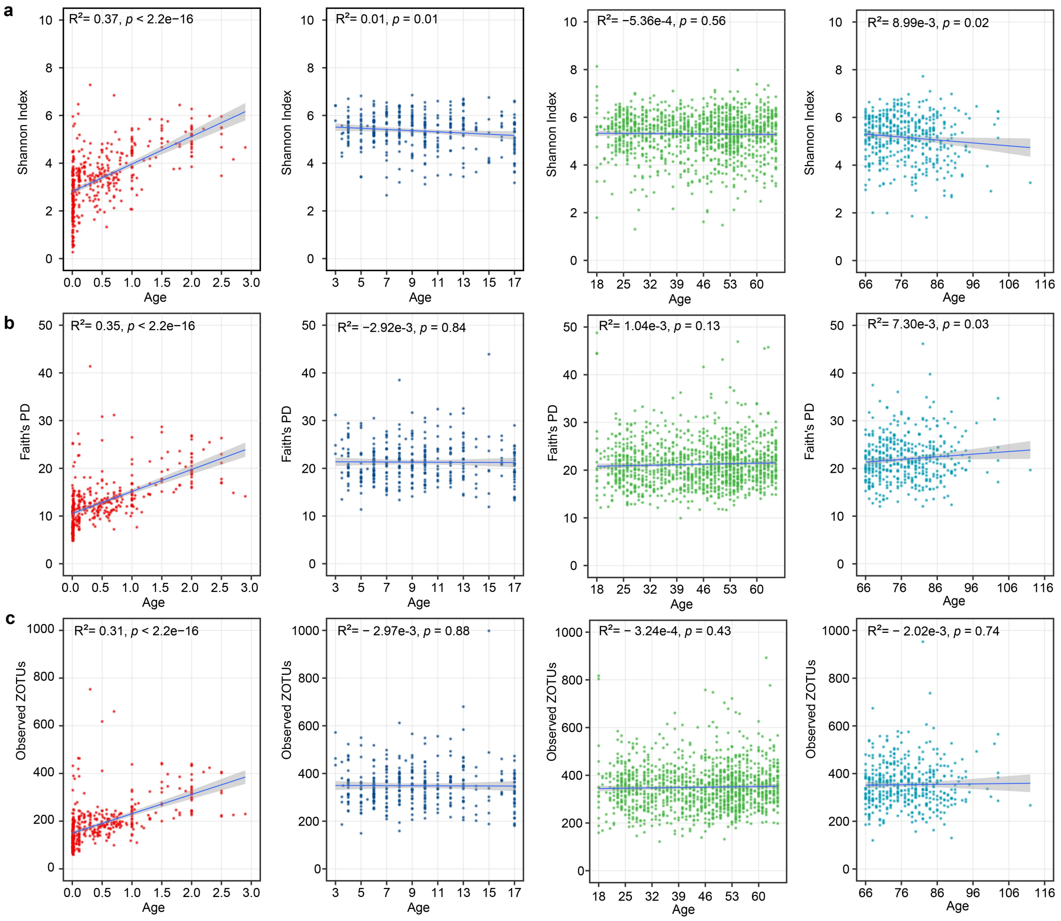

Supplementary Fig 1. The association between microbiota alpha diversity and age. The association between Shannon index **(a)**, Faith's PD **(b)**, Observed ZOTUs **(c)** and age groups of < 3, 3-17, 18-65 and 66-112 were shown.

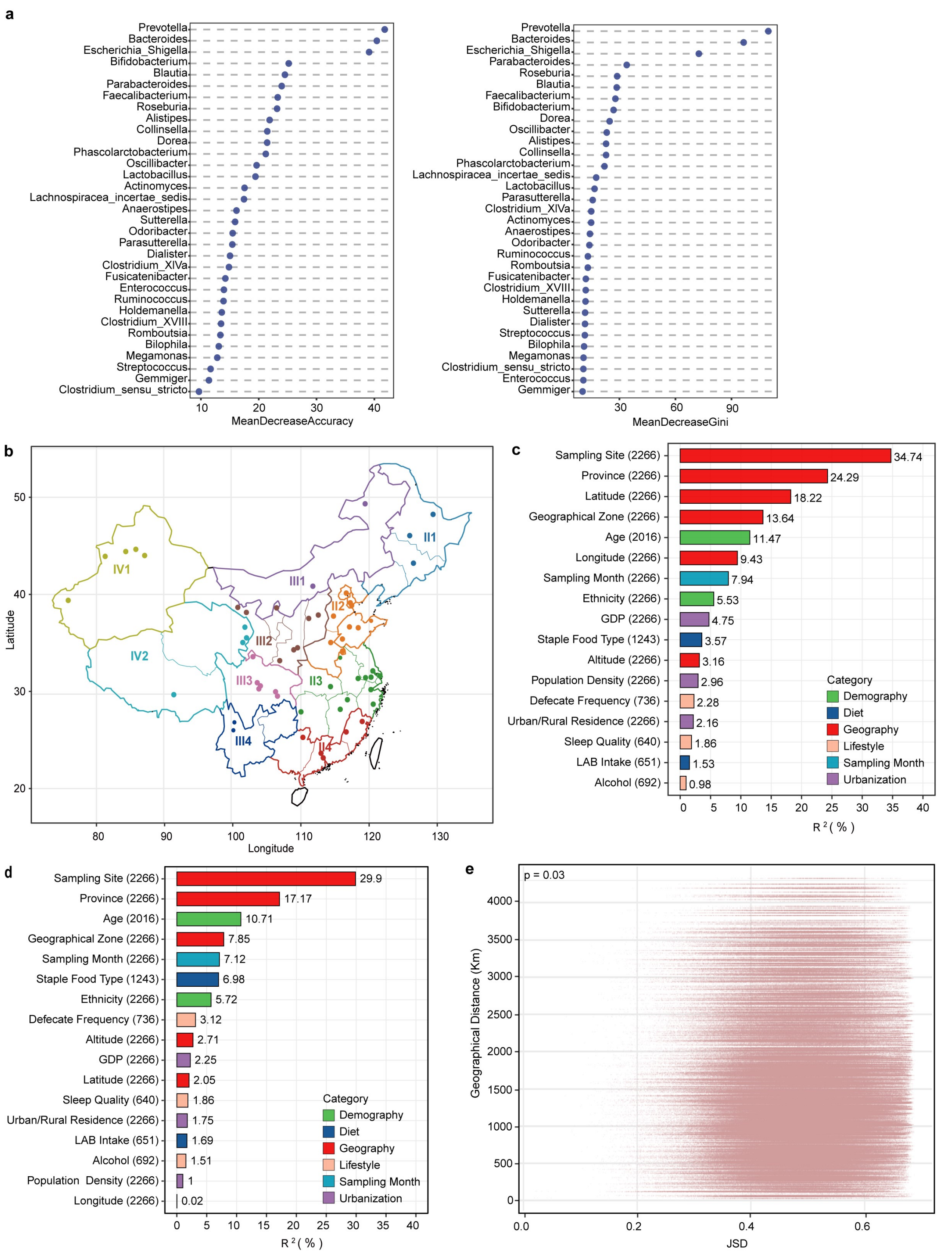

Supplementary Fig 2. **The gut microbiota composition of the Chinese population and associated covariates**. **(a)** Mean Decrease in Accuracy and Mean Decrease in Gini of genera from random forest models classifying enterotypes. **(b)** Map showing ten geographical zones of China. Each dot represents a sampling site. **(c-d)** Covariates associated with microbiota beta diversity estimated by Bray-Crutis distance (c) and Unweight UniFrac distance (d). The effect size was calculated with envfit (vegan), and covariates with FDR-corrected $p < 0.05$ were shown. The number of samples was indicated in brackets following each covariate. **(e)** Correlation between geographic distances and microbial JSD, estimated by Mantel statistic based on Spearman's rank correlation.

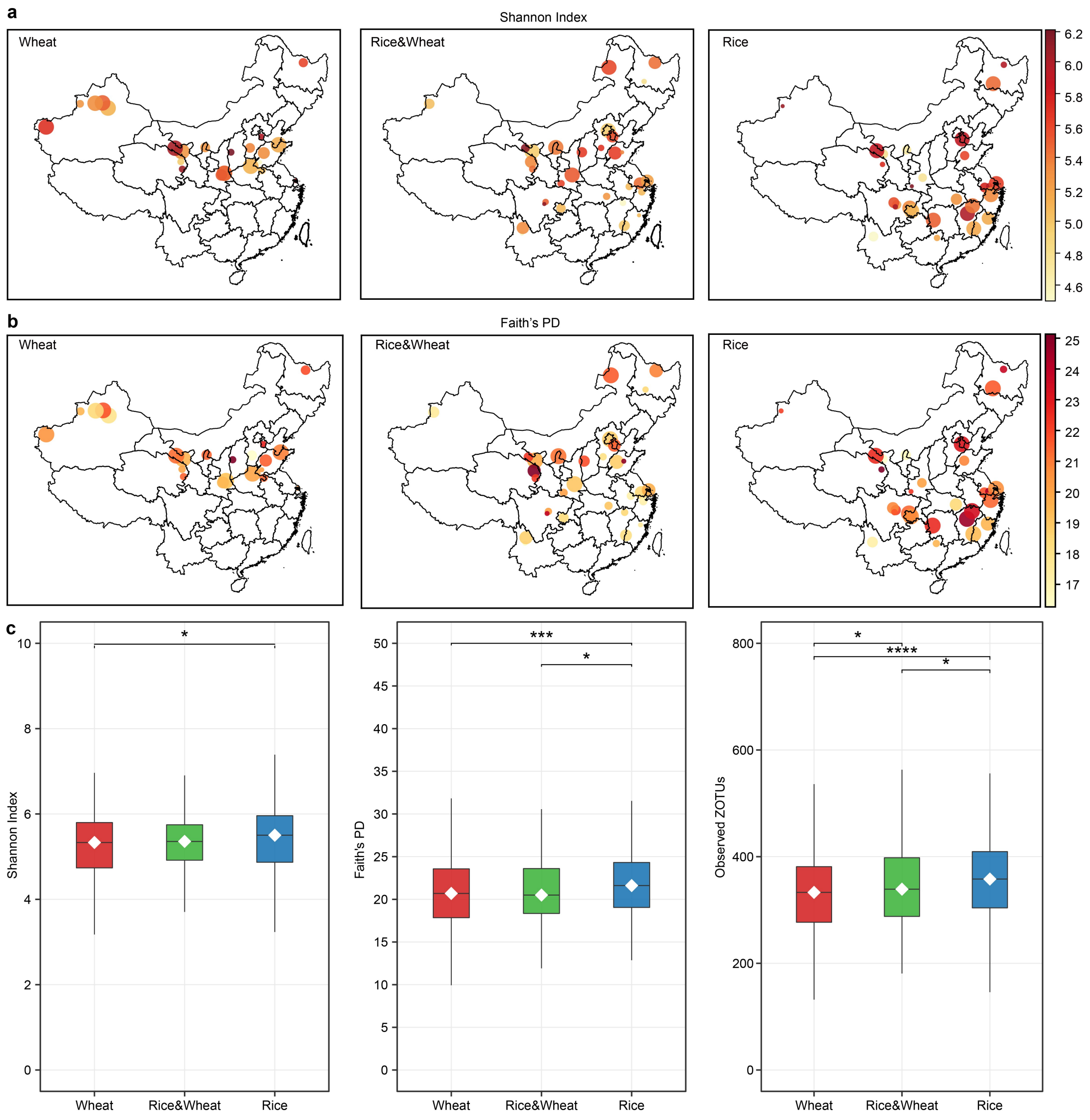

Supplementary Fig 3. Microbiota alpha diversity of populations consuming different staple food. **(a)** Shannon index. **(b)** Faith's PD. In a-b, each dot represents a sampling site; the color of dot indicates the median value in each site; the diameter of dot is proportional to the number of samples in each site and is fixed for sites with more than 15 samples. **(c)** Boxplot showing differences in alpha diversity indices between the Rice, Wheat and Rice & Wheat group. *$p < 0.05$, *** $p < 0.001$, Mann-Whitney test. In boxplots, the center line represents median, box limits represent upper and lower quartiles and whiskers represent 1.5x interquartile range.

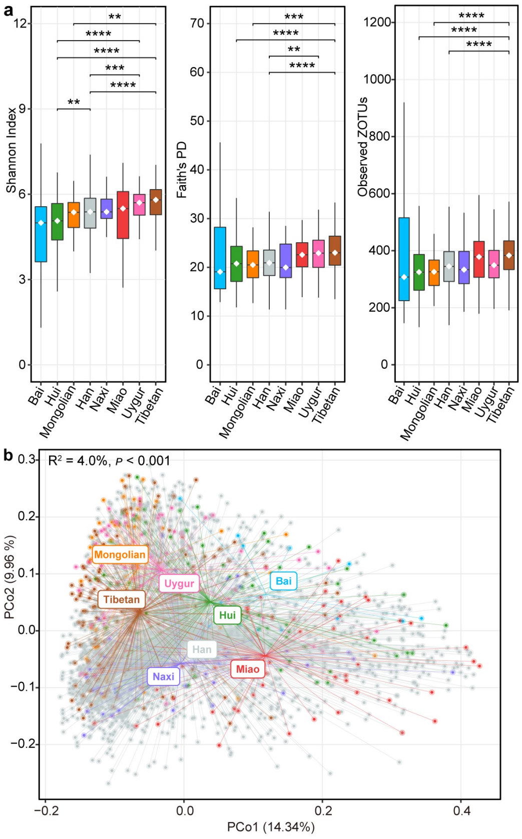

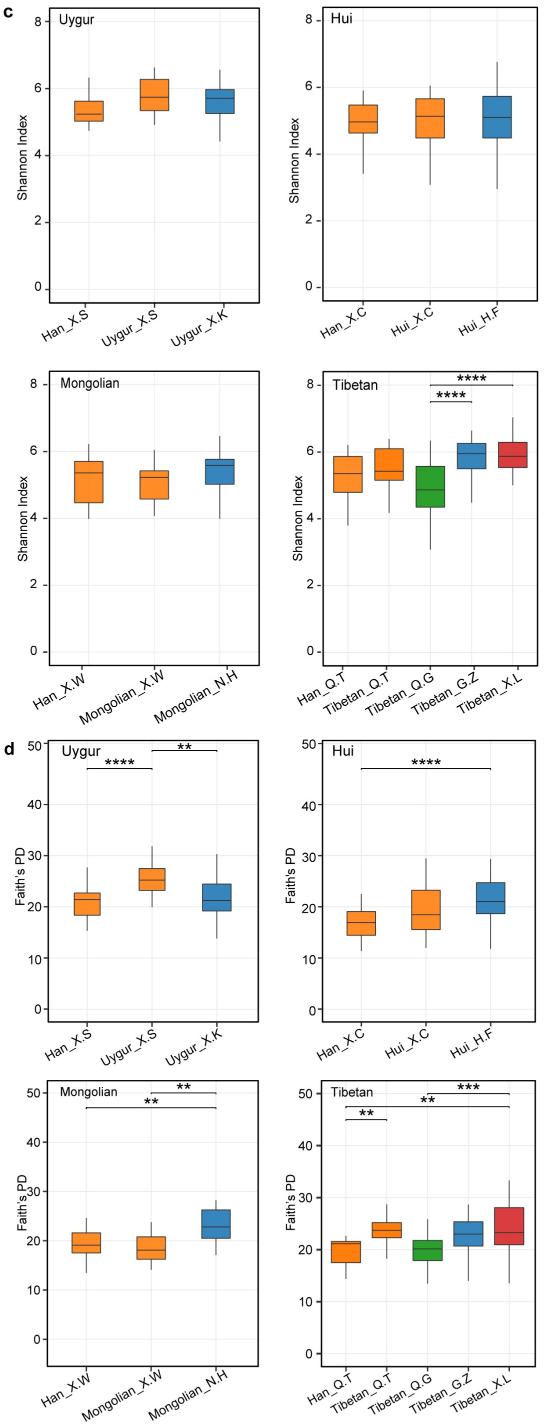

e

Ethnicity	Sensitivity	Specificity	Precision	AUC
Bai	0.29	0.99	0.68	0.93
Han	0.56	0.86	0.46	0.80
Hui	0.44	0.91	0.48	0.82
Miao	0.64	0.96	0.68	0.94
Mongolian	0.26	0.98	0.60	0.80
Naxi	0.33	0.99	0.82	0.91
Tibetan	0.88	0.81	0.62	0.92
Uygur	0.60	0.98	0.80	0.93

Supplementary Fig 4. **Gut microbiota diversity of different ethnic groups. (a)** Alpha diversity indices of different ethnic groups. ***p*.adj < 0.01, ****p*.adj < 0.001, *****p*.adj < 0.0001, Mann-Whitney test. **(b)** PCoA plot based on JSD. R^2 and *p* from the PERMANOVA test were shown. **(c-d)** Shannon index (c) and Faith's PD (d) of different ethnic groups per sampling site. The labels of x axis indicate ethnic groups followed by sampling sites. ***p*.adj < 0.01, ****p*.adj < 0.001, *****p*.adj < 0.0001, Mann-Whitney test. **(e)** Performance of random forest models distinguishing ethnic groups. The result was calculated as the average value from 1,000 random forest models. In boxplots, the center line represents median, box limits represent upper and lower quartiles and whiskers represent 1.5x interquartile range.

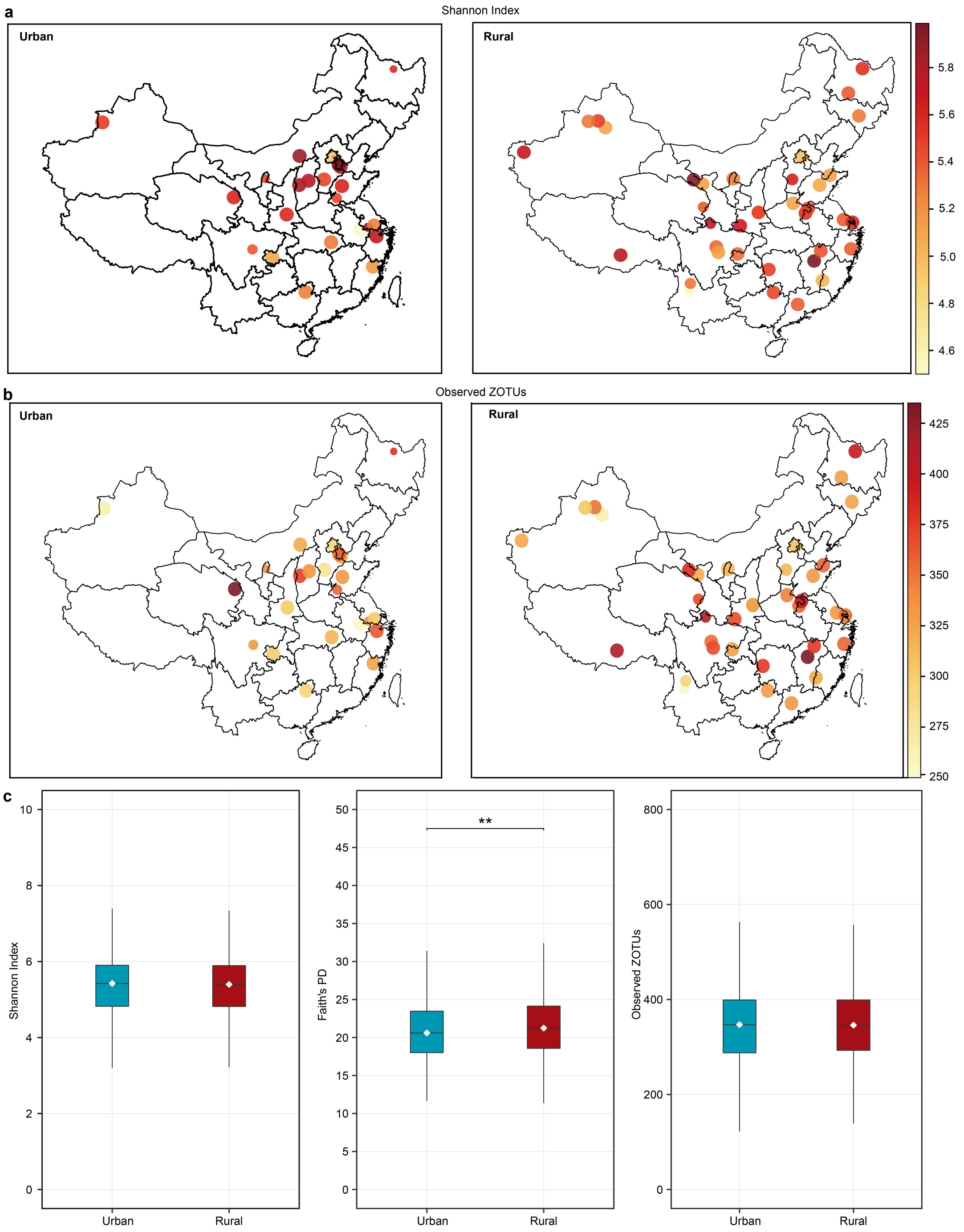

Supplementary Fig 5. Microbiota alpha diversity of urban and rural populations. (a) Shannon index. **(b)** Observed ZOTUs. In a-b, each dot represents a sampling site; the color of dot indicates the median value in each site; the diameter of dot is proportional to the number of samples in each site and is fixed for sites with more than 15 samples. **(c)** Boxplot showing differences in alpha diversity indices between urban and rural populations. $**p < 0.01$, Mann-Whitney test. In boxplots, the center line represents median, box limits represent upper and lower quartiles and whiskers represent 1.5x interquartile range.
