# Supplementary material for: Chinese gut microbiota and its associations with staple food type, ethnicity, and urbanization
Source: NPJ Biofilms Microbiomes. 2021 Sep 6;7:71. doi: 10.1038/s41522-021-00245-0 (PMC8421333; doi:10.1038/s41522-021-00245-0)
Supplement: Supplementary file 5 — Reporting Summary [file 41522_2021_245_MOESM5_ESM.pdf]

nature research

Corresponding author(s): Wenwei Lu, Mingkun Li and Wei Chen

Last updated by author(s): Jan 5, 2021

Reporting Summary

Nature Research wishes to improve the reproducibility of the work that we publish. This form provides structure for consistency and transparency in reporting. For further information on Nature Research policies, see our Editorial Policies and the Editorial Policy Checklist.

Statistics

For all statistical analyses, confirm that the following items are present in the figure legend, table legend, main text, or Methods section.

n/a	Confirmed	
☐	☒	The exact sample size (*n*) for each experimental group/condition, given as a discrete number and unit of measurement
☐	☒	A statement on whether measurements were taken from distinct samples or whether the same sample was measured repeatedly
☐	☒	The statistical test(s) used AND whether they are one- or two-sided<br>*Only common tests should be described solely by name; describe more complex techniques in the Methods section.*
☐	☒	A description of all covariates tested
☐	☒	A description of any assumptions or corrections, such as tests of normality and adjustment for multiple comparisons
☐	☒	A full description of the statistical parameters including central tendency (e.g. means) or other basic estimates (e.g. regression coefficient) AND variation (e.g. standard deviation) or associated estimates of uncertainty (e.g. confidence intervals)
☐	☒	For null hypothesis testing, the test statistic (e.g. *F*, *t*, *r*) with confidence intervals, effect sizes, degrees of freedom and *P* value noted<br>*Give P values as exact values whenever suitable.*
☒	☐	For Bayesian analysis, information on the choice of priors and Markov chain Monte Carlo settings
☒	☐	For hierarchical and complex designs, identification of the appropriate level for tests and full reporting of outcomes
☐	☒	Estimates of effect sizes (e.g. Cohen's *d*, Pearson's *r*), indicating how they were calculated

Our web collection on statistics for biologists contains articles on many of the points above.

Software and code

Policy information about availability of computer code

Data collection

No software was used for data collection.

Data analysis

STATA package v15.0 was used to calculate BMI z-scores.
USEARCH v11.0.667 was used to merge paired-end sequencing reads, cluster ZOTUs, and assign taxonomy of ZOTUs.
RDP Classifier v2.12 was used to classify the taxonomy of each sequence.
Cutadapt v2.11 was used to de-multiplex reads, remove barcodes and primers.
QIIME2 v2018.10 was used to construct phylogenetic tree, estimate Observed ZOTUs, Faith's PD, Shannon index, Bray-Curtis distance and Unweighted UniFrac distance.
phyloseq R package v1.32.0 was used to estimate JSD.
PICRUSt2 was used to predict functional potential.
vegan R package v2.5-6 was used to evaluate evenness index, microbiota variation explained by environmental variables, and perform PERMANOVA.
In enterotype analysis, cluster R package v2.1.0 was used to cluster samples, ade4 R package v1.7-15 was used to visualized the clustering on PCoA, and randomForest R package v4.6-14 was used to identify driving genera of each enterotype.
scikit-learn Python package v0.23.1 was used for the multiclass random forest models.
SparCC and Cytoscape v3.5.1 was used to construct the microbiota co-occurrence network.
DESeq2 R package v1.29.14 was used to detect differential genera.
Data analysis and graphical representations were performed using R (v4.0.2) and python (3.8.3).

For manuscripts utilizing custom algorithms or software that are central to the research but not yet described in published literature, software must be made available to editors and reviewers. We strongly encourage code deposition in a community repository (e.g. GitHub). See the Nature Research guidelines for submitting code & software for further information.

Data

Policy information about availability of data

All manuscripts must include a data availability statement. This statement should provide the following information, where applicable:

- Accession codes, unique identifiers, or web links for publicly available datasets
- A list of figures that have associated raw data
- A description of any restrictions on data availability

The microbial DNA sequences encoding 16S rRNA V3-4 region reported in this paper have been deposited in the Genome Sequence Archive in National Genomics Data Center, Beijing Institute of Genomics (China National Center for Bioinformation), Chinese Academy of Sciences, under accession number CRA003616 that are publicly accessible at https://bigd.big.ac.cn/gsa. The preview link is http://bigd.big.ac.cn/gsa/s/c6raNAiL.

Field-specific reporting

Please select the one below that is the best fit for your research. If you are not sure, read the appropriate sections before making your selection.

☒ Life sciences ☐ Behavioural & social sciences ☐ Ecological, evolutionary & environmental sciences

For a reference copy of the document with all sections, see nature.com/documents/nr-reporting-summary-flat.pdf

Life sciences study design

All studies must disclose on these points even when the disclosure is negative.

Sample size	To cover the diversity of Chinese gut microbiota as much as possible, a total of 3,183 participants were enrolled in the original study, and one fecal sample from each participant was collected. After sample exclusion as detailed below, 2,678 samples were used in the analysis.
Data exclusions	Samples were excluded if 1) the participant had gastrointestinal tract disorder or any other acute/chronic/recurrent medical conditions, 2) the participant had taken antibiotics in the three months prior to participation, 3) the sequencing reads were lower than 10,000.
Replication	To check the abundance of Bifidobacterium, 20 samples with different levels of Bifidobacterium were re-analyzed (from DNA extraction to sequencing data analysis) by another independent lab. The results showed a high consistency.
Randomization	Samples were allocated into groups based on their demographic features or associated environmental factors during data analysis, which did not require randomization.
Blinding	This study does not involve any treatment groups that would require blinding during assignment of subjects to groups.

Reporting for specific materials, systems and methods

We require information from authors about some types of materials, experimental systems and methods used in many studies. Here, indicate whether each material, system or method listed is relevant to your study. If you are not sure if a list item applies to your research, read the appropriate section before selecting a response.

Materials & experimental systems

n/a	Involved in the study	
☒	☐	Antibodies
☒	☐	Eukaryotic cell lines
☒	☐	Palaeontology and archaeology
☒	☐	Animals and other organisms
☐	☒	Human research participants
☒	☐	Clinical data
☒	☐	Dual use research of concern

Methods

n/a	Involved in the study	
☒	☐	ChIP-seq
☒	☐	Flow cytometry
☒	☐	MRI-based neuroimaging

Human research participants

Policy information about studies involving human research participants

Population characteristics	A total of 412 participants were under age 3. For the 2,266 participants with age 3-112, the median age was 46, including 1,144 males and 973 females. There were 1,755 Han Chinese and 487 individuals from seven ethnic minority groups, namely, Tibetan, Hui, Miao, Uygur, Naxi, Mongolia and Bai. All participants included in the analysis were generally healthy.
Recruitment	The 63 sampling sites from 28 provinces were chosen to represent different geographic conditions and urbanization status in China. Participants were randomly recruited from each sampling site.

Ethics oversight	The study was approved by the Ethical Committee of Jiangnan University. Written informed consents were obtained from all participants or their legal representatives for minors.

Note that full information on the approval of the study protocol must also be provided in the manuscript.
